# Post-Translational Modification and Natural Mutation of TRPC Channels

**DOI:** 10.3390/cells9010135

**Published:** 2020-01-07

**Authors:** Xianji Liu, Xiaoqiang Yao, Suk Ying Tsang

**Affiliations:** 1 School of Life Sciences, The Chinese University of Hong Kong, Hong Kong, China; liuxianji@link.cuhk.edu.hk; 2 School of Biomedical Sciences, The Chinese University of Hong Kong, Hong Kong, China; yao2068@cuhk.edu.hk; 3 State Key Laboratory of Agrobiotechnology, The Chinese University of Hong Kong, Hong Kong, China; 4 Key Laboratory for Regenerative Medicine, Ministry of Education, The Chinese University of Hong Kong, Hong Kong, China; 5 The Institute for Tissue Engineering and Regenerative Medicine (iTERM), The Chinese University of Hong Kong, Hong Kong, China

**Keywords:** transient receptor potential canonical channel, post-translational modification, natural mutation

## Abstract

Transient Receptor Potential Canonical (TRPC) channels are homologues of Drosophila TRP channel first cloned in mammalian cells. TRPC family consists of seven members which are nonselective cation channels with a high Ca^2+^ permeability and are activated by a wide spectrum of stimuli. These channels are ubiquitously expressed in different tissues and organs in mammals and exert a variety of physiological functions. Post-translational modifications (PTMs) including phosphorylation, N-glycosylation, disulfide bond formation, ubiquitination, S-nitrosylation, S-glutathionylation, and acetylation play important roles in the modulation of channel gating, subcellular trafficking, protein-protein interaction, recycling, and protein architecture. PTMs also contribute to the polymodal activation of TRPCs and their subtle regulation in diverse physiological contexts and in pathological situations. Owing to their roles in the motor coordination and regulation of kidney podocyte structure, mutations of TRPCs have been implicated in diseases like cerebellar ataxia (moonwalker mice) and focal and segmental glomerulosclerosis (FSGS). The aim of this review is to comprehensively integrate all reported PTMs of TRPCs, to discuss their physiological/pathophysiological roles if available, and to summarize diseases linked to the natural mutations of TRPCs.

## 1. Introduction to TRPC

The veil of transient receptor potential canonical (TRPC) channels was lifted by the search of mammalian homologs of the Drosophila TRP channel [1,2,3,4,5,6], the photoreceptor required for visual transduction. As indicated by the term “canonical”, the TRPC family was first identified within the whole TRP superfamily because of their highest homology to the Drosophila TRP channel [7].

This family is composed of seven members. Based on their sequences and properties, TRPC channels can further be divided into two subfamilies, with TRPC1/4/5 falling into one and TRPC3/6/7 into the other [8]. Studies have indicated that TRPC2 is a pseudogene in humans [9] and it will not be further discussed in this review. All TRPC channels share a common topology; they have six transmembrane domains (TM1-TM6) with a pore located between TM5 and TM6. Both the long N- and C-terminus are located intracellularly. N-terminus harbors several motifs like the ankyrin repeats, a coiled-coil domain, and a putative caveolin-binding region. C-terminus harbors the TRP signature motif, a calmodulin/inositol 1,4,5-trisphosphate (IP_3_) receptor-binding (CIRB) region, and a PDZ-binding motif (unique to TRPC4/5). All these motifs are essential for the multimerization and trafficking of TRPCs, and the interaction of auxiliary proteins with TRPCs as reviewed elsewhere [7].

A functional TRPC channel is formed by the association of four subunits. Depending on the composition, it can either be a homotetramer or a heterotetramer. TRPCs can heterogeneously multimerize not only with TRPCs but also with other TRP proteins. For instance, TRPC1-TRPP2 [10], TRPC1-TRPV6 [11], and TRPC1-TRPV4 [12] heterotetramers have been reported. Through different combinations, heterotetramers are conferred distinct biophysical properties from that of homotetramers to further meet diverse physiological requirements [13,14].

TRPCs are non-selective cation channels that are permeable to a multitude of monovalent and divalent cations, including Ca^2+^ [1,2,3,4,5,6]. Due to the importance of Ca^2+^ in different kinds of cellular activity, including gene expression, proliferation, differentiation, apoptosis, migration, secretion, and muscle contraction, TRPCs have received wide attention as reviewed elsewhere [15]. The ubiquitous expression of these channels in different types of cell, tissue, and organ circumstantially confirm their biological importance [16]. A substantial number of studies have been conducted to explore their functions under normal and pathological conditions and revealed their roles in cardiac hypertrophy [17], vasoconstriction [18], neointimal hyperplasia [19], angiogenesis [20], platelet activation [21], myoblast differentiation [22], salivary fluid secretion [23], motor coordination [24], podocyte dysfunction [25], and respiratory rhythm regulation [26]. All of these effects are mediated by the Ca^2+^ influx through these channels.

TRPCs may be the most elusive ion channels ever reported. In the early days when they were just discovered, some evidence implied that they are the potential store-operated calcium channels (SOCCs), but conflicting results and interpretations emerged later, making this identity controversial as reviewed elsewhere [27]. On one hand, there were findings showing that STIM and Orai are essential players of the store-operated calcium entry (SOCE) [28,29,30,31,32]. On the other hand, there were also studies showing TRPC channels are regulated by Orai [33] or STIM [34], thus conferring SOCC properties. The current view is that both Orai and TRPC would be activated by a decrease in ER [Ca^2+^] that is detected by the ER-located STIM. Although Ca^2+^ signals generated by both Orai and TRPC overlap with each other, they regulate distinct functions in different cells. A recent review has thoroughly examined this [35]. In fact, TRPCs are more commonly regarded as receptor-operated calcium channels (ROCCs). G protein-coupled receptors (GPCRs) on the plasma membrane sense the external stimuli such as hormones, neurotransmitters, and growth factors, leading to the activation of phospholipase C (PLC) through G protein; activated PLC catalyzes the conversion of phosphatidylinositol 4,5-bisphosphate (PIP_2_) to IP_3_ and diacylglycerol (DAG). TRPC3/6/7 can be directly activated by DAG, while TRPC1/4/5 are activated through more complicated pathways, interacting protein partners and phosphorylation may both be involved in this process [14,36,37,38]. It was found that scaffolding proteins such as the Na^+^/H^+^ exchanger regulatory factor (NHERF) binds to TRPC4/5 and desensitize these two channels to DAG. TRPC4/5 only would become sensitive to DAG when NHERF is dissociated from TRPC4/5, which can be caused by depletion of PIP_2_ or inhibition of protein kinase C (PKC) [36]. Apart from gating mechanisms like SOCCs and ROCCs, TRPC1, TRPC5, and TRPC6 were also reported to be mechanosensitive channels [39,40,41]. On the other hand, some evidence suggested that this property is conferred or potentiated by GPCRs [42,43].

Various signals input from GPCRs upstream of TRPCs and diverse biological effects triggered by Ca^2+^ influx downstream of TRPCs together make TRPCs the most versatile ion channels. These effects also increase the complexity of studies on TRPCs. To gain a better understanding of their behavior, plenty of studies have been carried out to explore the post-translational modifications (PTMs) and mutations of TRPCs. Both PTMs and mutations may modulate the properties of the channels under different conditions. Different kinds of PTM have been found in TRPCs; they include N-glycosylation, disulfide bond formation, ubiquitination, S-nitrosylation, S-glutathionylation, acetylation, and mostly, phosphorylation. These covalent modifications may change the activity, the subcellular location, or the protein-protein interaction of TRPCs, consequently modulating their function. Alterations of TRPC functions would further affect physiology and pathophysiology of different cells, tissues, and systems. Similarly important aspect is the natural mutations occurring in TRPCs. Natural mutations including deletion, frame shift, and substitution of TRPCs were reported; they result in gain-of-function or loss-of-function mutants that finally lead to inherited diseases. Here we try to compile all related reports of PTMs and mutations, aiming to provide an overview of the research in this field.

## 2. PTM of TRPC1

### 2.1. Phosphorylation-Induced Activation of TRPC1

In vascular endothelial cells, tumor necrosis factor α (TNFα) promotes TRPC1 expression via the NF-𝜅B-dependent pathway, increasing Ca^2+^ influx and endothelial permeability [44,45,46]. The influence of TRPC1 is determined not only by its expression level but also by its activity. Thrombin stimulation evokes protein kinase C (PKCα) to phosphorylate TRPC1, as a result, augments TRPC1-mediated Ca^2+^ entry and endothelial permeability [47]. Interestingly, TRPC1 phosphorylation-induced hyper-permeability of confluent endothelial monolayer is reported to be a reason underlying the vascular toxicity of platinum-based chemotherapy [48]. Apart from its role in endothelial cells, TRPC1-mediated SOCE is suggested to be responsible for vasoconstriction in different types of vascular smooth muscle cells [18,49,50]. Saleh et al. suggested that depletion of sarcoplasmic reticulum (SR) [Ca^2+^]_i_ store caused PIP_2_ to directly bind to and subsequently activate TRPC1 in native rabbit portal vein myocytes. Furthermore, protein kinase C (PKC)-induced phosphorylation of TRPC1 was reported to be required for this PIP_2_ activation. The study highlighted the essential role of PIP_2_ and phosphorylation in the activation of TRPC1 in a SOCE-dependent manner [51]. In a later study, Shi et al. further explored the relationship between PKC and TRPC1. They found that in rabbit vascular smooth muscle cells, Ca^2+^ depletion in SR induced TRPC1-Gαq-PLCβ protein complex formation; PKC was sequentially activated in a classical PLC-dependent pathway, which in turn phosphorylated TRPC1 and enhanced the channel’s activity [52]. A follow-up study by the same group clearly indicated the involvement of STIM1 in coupling SR Ca^2+^ store depletion to this activation pathway involving TRPC1-Gαq-PLCβ [53]. In rat insulinoma cell line INS-1E, phosphorylation of TRPC1 by PKCα was also shown to increase the channel activity and promote the glucose-stimulated insulin secretion [54].

### 2.2. Phosphorylation-Induced Inhibition of TRPC1

Phosphorylation of TRPC1 has been investigated for a long time and been documented in many research articles. However, only few studies identified the exact modified sites. Employing TRPC1 oligopeptides that contain PKG phosphorylation sites, Zhang et al. successfully identified two phosphorylation sites of TRPC1, S172, and T313. Phosphorylation of TRPC1 via the NO-cGMP-PKG pathway downregulated the activity of TRPC1-TRPV4 heterotetramer in porcine coronary artery smooth muscle cells, which in turn attenuated 11,12-EET (a major type of physiological epoxyeicosatrienoic acid)-induced hyperpolarization and relaxation of smooth muscle [55]. This inhibitory modification of TRPC1 also provided important negative feedback which helped to fine tune the activity of TRPC1-TRPV4 heterotetramer in rat primary mesenteric artery endothelial cells [56].

### 2.3. PTM of TRPC1 Discovered by High-Throughput Experiments 

**Figure 1:**
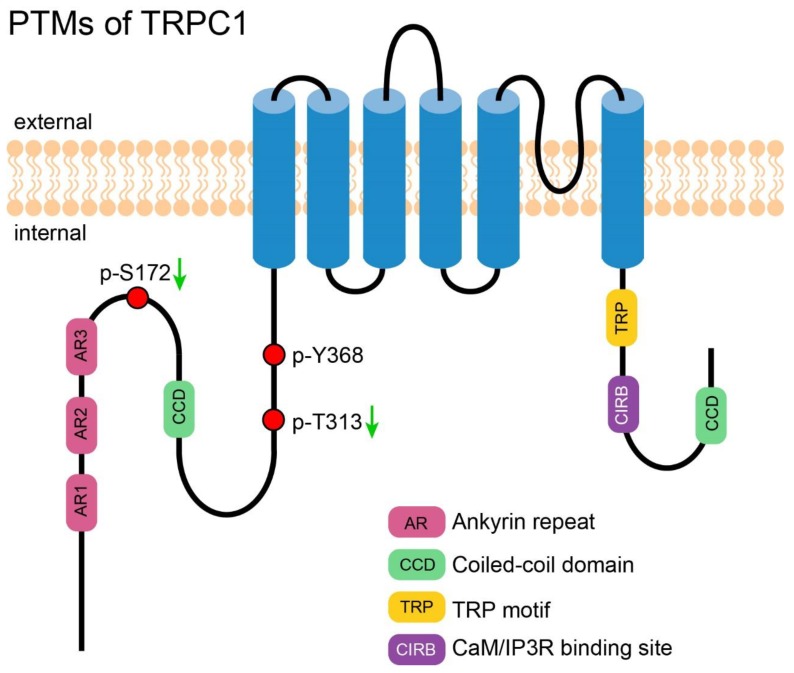
PTMs of TRPC1. A cartoon depicting TRPC1 with its important motifs and Post-translational modifications (PTMs—those exact positions had been reported). PTMs are presented in circles. Red circles represent the reported phosphorylation sites of TRPC1, which are labeled in the format of a “p-amino acid-position”. The green arrows near the labels denote the PTMs decreasing the channel’s activity.

Li et al. reported that the tyrosine Y368 of TRPC1 is phosphorylated in Hep3B cells, a non-metastatic hepatocellular carcinoma cell line, but not in MHCC97H, a highly metastatic hepatocellular carcinoma cell line [57]. This finding was intriguing and hinted that change of phosphorylation of TRPC1 may contribute to the metastasis of hepatocellular carcinoma. Figure 1 shows an overview of all PTMs of TRPC1.

## 3. PTM of TRPC3

### 3.1. N-Glycosylation of TRPC3

N-linked glycosylation of TRPC3 in the first extracellular loop (E1) was identified by Vannier et al. in 1998 [58]. Although functional study of this PTM was lacking, as inferred from a study of TRPC6 [59], N-linked glycosylation may serve to decrease TRPC3 basal activity. This study showed that TRPC6, a close relative of TRPC3, is dually glycosylated at E1 and a second extracellular loop (E2), respectively, while TRPC3 is monoglycosylated at E1 as described above. The TRPC3 channel exhibited a higher basal activity than TRPC6. Interestingly, deletion of the one glycosylation site increased and deletion of both further increased the TRPC6 activity [59]. In addition, engineering of an additional second glycosylated site to TRPC3 reduced its basal activity. Altogether, glycosylation of TRPC3 may serve to decrease the basal activity of TRPC3.

### 3.2. Phosphorylation-Induced Activation of TRPC3

The Src family of tyrosine kinase (STK) was previously demonstrated to play an obligatory role in the phosphorylation and activation of TRPC3 [60]. Based on this hint, Kawasaki et al. successfully discovered four phosphorylated tyrosine sites, Y49, Y148, Y150, and Y226, on TRPC3, but only phosphorylated-Y226 was essential for TRPC3 activation. The authors also showed it was Src but not its close relatives Yes and Fyn that performed this modification. Interestingly, although Y226 is conserved between TRPC3 and TRPC6/7, the cognate tyrosine in TRPC6/7 do not affect the channels’ activity [61]. 

### 3.3. Phosphorylation-Induced Inhibition of TRPC3

T11 and S263 of TRPC3 were identified as PKG-catalyzed phosphorylation sites by Kwan et al. In TRPC3- and PKG-co-transfected HEK-293 cells, the phosphorylation led to a decline of the channel activity [62]. That was the first evidence supporting the idea that activated TRPC3 in the SOCE mode could be directly regulated by phosphorylation [62]. S712 of TRPC3, a highly conserved serine exists in all members of TRPC family, was also reported to be phosphorylated by PKC in HEK-293 cells; this phosphorylation decreased the channel activity stimulated by DAG analog 1-oleoyl-2-acetylsn-glycerol (OAG). Since DAG was also the agonist of PKC, Trebak et al. suggested a dual role of DAG in positive and negative regulation of TRPC3 [63]. Later, Kwan et al. showed that PKC-induced TRPC3 inhibition was partially mediated by PKG, suggesting that PKG regulates not only SOCE but also ROCE mediated by TRPC3 [64]. 

### 3.4. Phosphorylation-Mediated Protein-Protein Interaction of TRPC3

Phosphorylation at T573 of TRPC3 was shown to be obligatory to recruit immunophilin FKBP12, which in turn facilitates the formation of TRPC3-calcineurin complex and activation of the transcription factor NFAT. PKC, the kinase that catalyzes this phosphorylation, played an important role in coupling the Ca^2+^ which permeated through TRPC3 to the NFAT signaling pathway [65].

### 3.5. PTM of TRPC3 Discovered by High-Throughput Experiments

**Figure 2:**
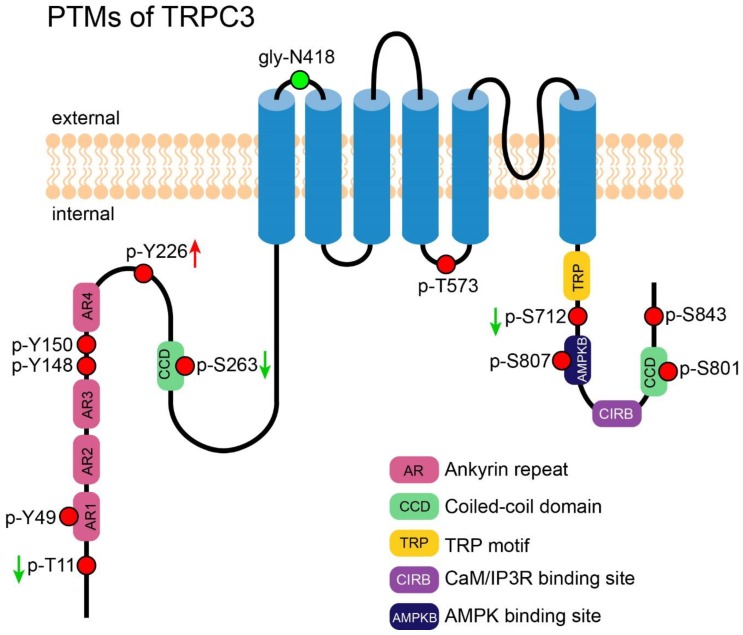
PTMs of TRPC3. A cartoon depicting TRPC3 with its important motifs and PTMs (those exact position had been reported). PTMs are presented in circles. Red circles represent the reported phosphorylation sites of TRPC3, which are labeled in the format of a “p-amino acid-position”. The green circle represents the reported N-glycosylation site; it is labeled in the format of a “gly-amino acid-position”. The green arrows near the labels denote the PTMs decreasing the channel’s activity. The red arrows near the labels denote the PTMs which increase the channel’s activity.

Several novel phosphorylation sites were reported from large-scale mass spectrometry-based experiments, for instance, S56 and S807 were identified from mouse brain [66,67], while S801 was detected in rat intestine [68]. Phosphorylation on S843 was found in Hela cells and the phosphorylation decreased after treatment of rapamycin, an inhibitor of mammalian target of rapamycin (mTOR) [69]. However, the biological significance of these phosphorylation is yet to be determined. Figure 2 shows an overview of all PTMs of TRPC3.

## 4. PTM of TRPC4

### 4.1. Phosphorylation-Mediated Activation of TRPC4

STK-dependent phosphorylation on Y959 and Y972 in response to the stimulation of epidermal growth factor (EGF) enhanced the association of TRPC4 with NHERF. NHERF facilitated the exocytotic insertion of TRPC4 into plasma membrane, leading to an increased TRPC4-mediated Ca^2+^ influx [70]. In TRPC4-overexpressed HEK-293 cells, application of the phosphodiesterase (PDE) 5 inhibitor increased TRPC4 activity by enhancing cGMP-dependent PKG activity, which then phosphorylated S688 of the channel [71]. Although TRPC4 is characterized as ROCC, it cannot be activated by DAG directly unless PIP_2_ is depleted. Storch et al. proved that PIP_2_ depletion caused a conformational change of TRPC5 and a dissociation of NHERF from TRPC5, making this channel sensitive to DAG. Phosphorylation at T972 of TRPC5 was proved to be necessary for the binding of NHERF [36]. Although it is not studied directly, T971 of TRPC4 is homologous to T972 of TRPC5; both residues are located in the PDZ binding motif, which is the binding site of NHERF. So, it is possible that T971 of TRPC4 has a similar effect.

### 4.2. PTM of TRPC4 Discovered by High-Throughput Experiments

By using immunoprecipitation-coupled mass spectrometry, Lee et al. launched the first global search of potential phosphorylation sites on TRPC4. They found three novel phosphorylated residuals: T691, S875, and T879 [72]. Phosphorylation on S193 was identified in Hela [73] and S955 was discovered in liver [74] through mass spectrometry, however, there is no functional annotation on these phosphorylated sites.

### 4.3. Ubiquitination of TRPC4

TRPC4 is so far the only member in the TRPC family that has been reported to undergo ubiquitination [75]. In the presence of AIP4 (itchy E3 ubiquitin protein ligase), TRPC4 was strongly ubiquitinated in HEK-293 cells. Instead of inducing degradation, AIP4-mediated ubiquitination led to internalization and accumulation of the channel in the intracellular compartment. It had been shown that the ubiquitination did not shorten the channel’s half-life time but instead increased its steady-state level, meaning the internalized channel could be targeted to plasma membrane again. The significance of such internalization may lie in reducing cytotoxicity caused by the ion channel proteins, as they may be less harmful to be located inside the cell than to be located on the plasma membrane. This study of ubiquitination of TRPC4 provides insights into the recycling mechanism of these channels.

### 4.4. Disulfide Bond Formation in TRPC4

**Figure 3:**
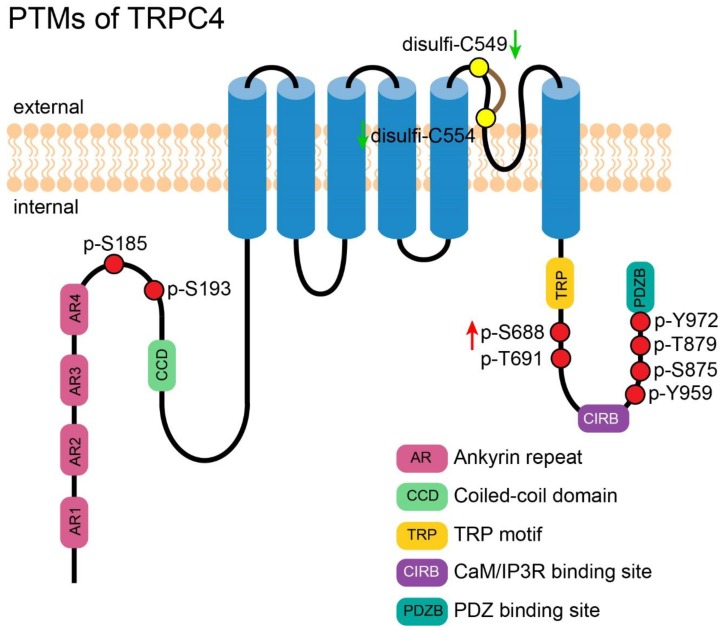
PTMs of TRPC4. A cartoon depicting TRPC4 with its important motifs and PTMs (those exact positions had been reported). PTMs are presented in circles. Red circles represent the reported phosphorylation sites of TRPC4, which are labeled in the format of a “p-amino acid-position”. Yellow circles represent the reported residues for disulfide bond formation, which are labeled in the format of a “disulfi-amino acid-position”. The green arrows near the labels denote the PTMs decreasing the channel’s activity. The red arrows near the labels denote the PTMs which increase the channel’s activity.

A disulfide bond was reported to form between C549 and C554 in TRPC4, though a reduction of this bond by DTT or TECP augmented the TRPC4 whole cell current. Double mutations C549A and C554A disabled the activation by DTT but the channel could still be activated by the TRPC4 activator englerin A. Surprisingly, single mutation of either C549A or C554A totally abrogated the response of TRPC4 to either englerin A or DTT, suggesting the pore loop architecture was severely damaged, so it lost the ability to be activated exogenously. In addition, the results suggested the importance of a disulfide bond in stabilizing the architecture of a channel’s pore [76]. Figure 3 shows an overview of all PTMs of TRPC4.

## 5. PTM of TRPC5

### 5.1. Phosphorylation of TRPC5

TRPC5 was desensitized quickly after activation by GPCR, and PKC-mediated phosphorylation at T972 was revealed to underpin this modulation. Introducing a T972A mutation into TRPC5 greatly slows down the process [77]. This finding established a new function of phosphorylation, as it may modulate the kinetics of an ion channel. Later in 2017, Storch et al. found that phosphorylation at T972 of TRPC5 was necessary for the binding of NHERF to this channel. Binding of NHERF abolished the activation of TRPC5 by DAG. Introducing a T972A mutation into TRPC5 interrupted the interaction between TRPC5 and NHERF and made TRPC5 sensitive to DAG [36]. Sung et al. reported that TRPC5 could be phosphorylated by PKA via a Gs/cAMP/PKA signaling pathway at S794 and S796, and subsequently decreased its activity [78].

### 5.2. Disulfide Bond Formation in TRPC5

Formation of disulfide bond between C553 and C558, two residues located at the third extracellular loop (E3) near the pore of TRPC5, was first reported by Xu et al. in 2008 [79]. Breaking the disulfide bridge by extracellular reduced thioredoxin (rTRX) activated TRPC5 homotetramer and TRPC5/1 heterotetramer in secretory fibroblast-like synoviocytes and suppressed synovial fluid secretion, leading to rheumatoid arthritis. Elaborate functional study of the disulfide bond in TRPC5 was done by Hong et al. in 2015 [80]. They found that double mutations C553S and C558S completely abolished lanthanides- or receptor-mediated activation of TRPC5, suggesting that the disulfide bond is necessary for the channel activation. The TRPC5 mutant showed a weaker interaction with wild-type TRPC5 and the decreased plasma expression. The results suggested that the disulfide bond is important for the multimerization and plasma membrane trafficking of TRPC5 [81]. 

### 5.3. S-Nitrosylation of TRPC5

Apart from formation of the disulfide bond, C553 and C558 of TRPC5 were also reported to be the sites of nitric oxide (NO)-induced S-nitrosylation. This sensitivity to NO provides an alternative activation mechanism apart from the receptor-operated mode. NO directly modifies free sulfhydryl groups, inducing S-nitrosylation on these two cysteines, which causes a bend of TM6 and opens the gate of the channel. Ca^2+^-dependent NO synthases (NOSs) are responsible for the generation of NO. Ca^2+^ influx through TRPC5 activates NOSs to produce NO; the increased NO in turn enhances the TRPC5-elicited Ca^2+^ mobilization, and therefore helps in building up a positive feedback cycle. With this positive feedback mechanism, TRPC5 distributed in an endothelial cell layer of vascular tissue may be involved in amplifying the effect of NO to synchronize the neighboring smooth muscle cells in vascular relaxation [82]. However, inconsisent findings were reported by Wong et al. [83]. The authors reported that S-nitroso-N-acetylpenicillamine (SNAP) at a concentration that is sufficient to generate NO and cause vasodilation failed to activate or inhibit exogenously-expressed TRPC5 in HEK-293. Although Wong et al. reported that they occasionally observed a stimulatory effect of SNAP, this occurred only at a concentration higher than that necessary to trigger NO-dependent effects and the effect was not statistically significant. Moreover, SNAP was found to inhibit endogenous Ca^2+^ entry in bovine aortic endothelial cells (BAECs). Wong et al. concluded that NO was not a direct modulator of TRPC5; instead of activating TRPC5, NO may inhibit TRPC5 in BAECs that endogenously express the channels. The authors suggested that C553 and C558 may not be the sites for S-nitrosylation without reducing reagents since they are occupied by a disulfide bond. It is difficult to reconcile such a discrepancy, but prevalent literatures have shown that NO is a negative regulator suppressing Ca^2+^ entries in endothelial cells [84,85,86].

### 5.4. S-Glutathionylation of TRPC5

**Figure 4:**
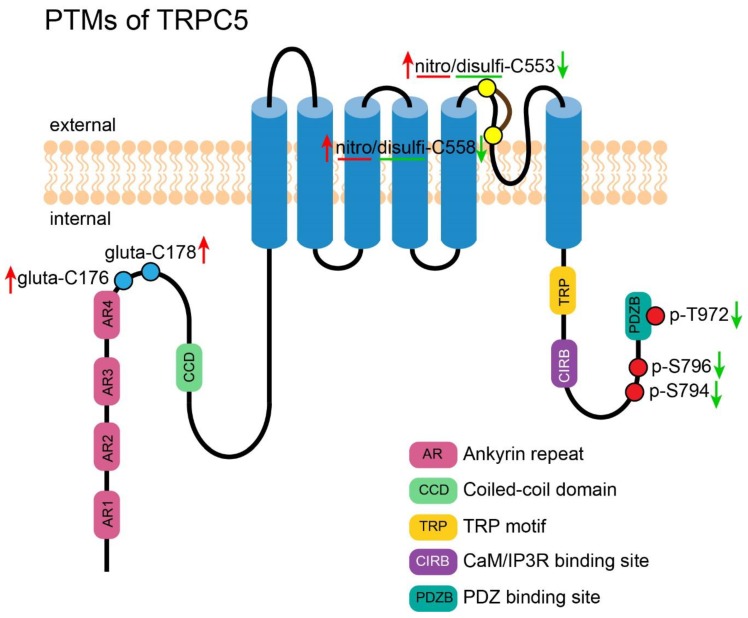
PTMs of TRPC5. A cartoon depicting TRPC5 with its important motifs and PTMs (those exact positions had been reported). PTMs are presented in circles. Red circles represent the reported phosphorylation sites of TRPC5, which are labeled in the format of a “p-amino acid-position”. Yellow circles represent the reported residues for S-nitrosylation and disulfide bond formation, which are labeled in the format of a “nitro/disulfi-amino acid-position”. Blue circles represent the reported residues for S-glutathionylation, which are labeled in in the format of a “gluta-amino acid-position”. The green arrows near the labels denote the PTMs decreasing the channel’s activity. The red arrows near the labels denote the PTMs which increase the channel’s activity.

C176 and C178 of TRPC5 were shown to be glutathionylated by intracellular oxidized glutathione (GSSG) in striatal neurons. TRPC5 S-glutathionylation caused a sustained elevation of cytosolic Ca^2+^, activated a calmodulin-dependent protein kinase and calpain-caspase pathway, and subsequently led to the death of striatal neurons. In the striatum of both transgenic mice and patients with Huntington’s disease, increased glutathionylated-TRPC5 level was detected; this was suggested to underlie the neurodegeneration in Huntington’s disease. Indeed, decreasing the activity of TRPC5 by either knockdown strategy or pharmacological inhibition reduced striatal neuronal cell death induced by oxidation. Moreover, application of a TRPC5 inhibitor in Huntington’s disease transgenic mice improved their rearing behavior. This study exploited the S-glutathionylation of TRPC5 that responds to oxidative stress and underlined its importance in the pathogenesis of Huntington’s disease [87]. Figure 4 shows an overview of all PTMs of TRPC5.

## 6. PTM of TRPC6

### 6.1. Glycosylation of TRPC6

TRPC6 is the best studied channel in the TRPC family in terms of PTMs. The first reported PTM of TRPC6 was glycosylation by Boulay et al. in 1997 when the mouse TRPC6 was cloned [6]. The glycosylation was confirmed by deglycosylation treatment and Western blotting. Two putative N-glycosylation sites locating at N472 and N560 (N473 and N561 in human, respectively) were suggested. Zhang et al. later cloned three distinct rat TRPC6 isoforms (rTRPC6a/b/c) and identified a putative N-glycosylation site at N711 (rTRPC6a), which is probably located at E3 near the pore. Both rTRPC6a and rTRPCb could be glycosylated but rTRPC6c, which lacks 68 amino acids locating at the cytoplasmic side immediately adjacent to TM6, could not be glycosylated. This short 68 amino acid segment was thus suggested to be important for the proper processing of TRPC6 including glycosylation [88]. TRPC6 exhibits lower constitutive activity than TRPC3 as evaluated by single channel recordings, and glycosylation was reported to account for this difference [59]. TRPC6 favors a dual N-linked glycosylation at N473 and N561, which are located at E1 and E2, respectively, while TRPC3 is monoglycosylated at E1. Introducing an N561Q mutation alone is sufficient to convert TRPC6 from a tightly regulated channel into a constitutively active one. Regulation of channel activity by glycosylation was further confirmed by reversely adding an extra N-glycosylation site to the E2 of TRPC3. This extra N-glycosylation site decreased the basal activity of TRPC3. It is noticeable that according to Dietrich et al.’s report, glycosylation decreased the activity of TRPC6 directly but did not affect its insertion into the plasma membrane. Gain-of-function (GOF) mutations of human TRPC6 (R895C) that lead to focal segmental glomerulosclerosis (FSGS) were found to be cytotoxic in different cultured cells, including podocytes. Mutations of two glycosylation sites at N473 and N561 reduced the plasma membrane expression of TRPC6 (of both the wild-type TRPC6 and the mutant TRPC6) and abrogated TRPC6 mutant-mediated cytotoxicity [89]. The discrepancy between Dietrich et al. and Talbot et al. in terms of the effect of N-glycosylation on TRPC6’s plasma membrane trafficking may be explained by the different assays employed in these two studies. Microscopy was used by Dietrich et al. to observe the subcellular location of the glycosylated TRPC6 in the cell, while cell surface biotinylation followed by pulldown using streptavidin-agarose beads and subsequent Western blotting was adopted by Talbot et al. to ascertain the plasma membrane insertion of glycosylated TRPC6. Due to the limitation of resolution and the number of cells examined, microscopy may be less sensitive to the potential changes. In Talbot et al., it was concluded that N-glycosylation at N473 and N561 is important for the membrane trafficking of the TRPC6 protein. Although most studies of glycosylation were carried out by the ectopic expression system, native N-glycosylation was also found in pulmonary vascular smooth muscle cells (PASMC) where TRPC6 was shown to regulate cell proliferation [90]. 

### 6.2. Acetylation of TRPC6

An organ-wide atlas of lysine acetylation sites in rat was established by Olsen and co-workers, where two acetylation sites of TRPC6 were found, with K170 detected in muscle while K370 in brain, heart and stomach [91]. Lysine acetylation had been intensively studied in the field of histone modulation. Although a number of papers have revealed this PTM in lots of other proteins, its function still remains ill-elucidated. Reports on acetylation in ion channels are scarce. Hancock et al. reported that acetylation decreased channel conductance and altered selectivity of protein P, an anion-specific ion channel from the outer membrane of *Pseudomonas aeruginosa* [92]. Acetylation of voltage-gated K^+^ channel Kv2.1 led to internalization of the channel and attenuated apoptosis in INS-1 β-cells [93]. According to the report by Butler et al., acetylation of epithelial Na^+^ channel elevated the channel abundance and plasma membrane expression by antagonizing ubiquitination and protein degradation [94]. Blockage of acetylation on K74 in voltage-dependent-anion channel was reported to decrease sperm motility [95]. Based on this scarce literature, acetylation is involved in the regulation of conductance, selectivity, trafficking, and turnover of ion channels and subsequently exerts different physiological functions. Acetylation of TRPC6 may be a strategy adopted by different cell types to fine tune the channel property and to meet distinct physiological demands. Further investigation in this area is needed.

### 6.3. Phosphorylation-Induced Activation of TRPC6

Hisatsune et al. was the first to document the phosphorylation of TRPC6 by Fyn. Fyn was reported to physically bind to TRPC6, phosphorylate the channel and increase its activity, although a specific modification site was not identified in this report [96]. T487 located at the E1 of TRPC6 was shown to undergo phosphorylation by Ca^2+^/calmodulin (CaM)-dependent kinase II (CaMKII); this phosphorylation was shown to potentiate the channel’s conductance. The modification may take place under basal status or after TRPC6 activation. The former was reported to sensitize TRPC6, while the latter was reported to increase [Ca^2+^]_i_ and activate CaMKII, which in turn boosts TRPC6 activity. This self-stimulation process is an important positive feedback mechanism of TRPC6 [97]. Through cAMP-PI3K-PKB-MEK-ERK1/2 signal transduction pathway, ERK was also found to phosphorylate TRPC6 at S281 to activate the channel. This phosphorylation may underlie the [Ca^2+^]_i_ increase in glomerular mesangial cells induced by glucagon, which promotes cell growth and proliferation, leading to glomerular injury [98].

### 6.4. Phosphorylation-Induced Inhibition of TRPC6

In neonatal rat cardiomyocytes, angiotensin II (Ang II) enhanced Ca^2+^ influx through TRPC3/6, leading to the activation of calcineurin/NFAT signaling pathway and subsequently causing cardiac hypertrophy [99]. Application of atrial and brain natriuretic peptides ameliorated cardiac hypertrophy through synthesis of cGMP. cGMP activated cGMP-sensitive-protein kinase G (PKG), leading to the phosphorylation of T69 (T70 in human) and S322 of TRPC6 to downregulate the channel’s activity and prevent excessive Ca^2+^ influx [100,101,102]. PDE5 inhibitor tadalafil is an antihypertrophic reagent entering clinical trial as a candidate to cure Duchenne muscular dystrophy. Application of tadalafil in dogs with golden retriever muscular dystrophy delays the onset of dystrophic cardiomyopathy. A study has shown that tadalafil decreased TRPC6 expression levels as well as permeation of Ca^2+^ by increasing the overall tyrosine phosphorylation of the channel in heart [103]. T69 phosphorylation of TRPC6 was also reported in vascular smooth muscle cells. Takahashi et al. have demonstrated that phosphorylation negatively regulated TRPC6 via NO–cGMP–PKG pathway, and proposed its physiological significance in maintaining local blood flow and lowering blood pressure [86]. Instead, protein kinase A (PKA) was documented to modify the same site in rat aortic smooth muscle cells. Phosphorylation caused by pretreatment of cilostazol, a specific PDE3 inhibitor which inactivated TRPC6 and attenuated vasoconstriction triggered by Ang II [104]. A partially conflicting report was made by Horinouchi et al. Although both S28 and T69 were phosphorylated by PKA through the adenylate cyclase/cAMP/PKA signaling pathway, only S28 but not T69 decreased TRPC6 activity [105]. T69 was confirmed to be the target of another enzyme cGMP-dependent protein kinase I (cGKI) in microcirculatory endothelial cells. This modification decreased TRPC6 activity and Ca^2+^ influx, while counteracting the hyperpermeability effects of histamine, which is a major part of atrial natriuretic peptide’s anti-inflammatory effect [106]. Using alanine screening of all predicted PKC sites, Bousquet et al. discovered a new phosphorylation site at S448. Phosphorylation of this serine residue in TRPC6 results in a decline of the channel’s activity. Here TRPC6 contributed the major component of vasopressin-induced cation current in A7r5 cells, while PKC acted as an important negative regulator to decrease Ca^2+^ entry through TRPC6 [107]. It was also reported that dysmotility of primary podocytes evoked by Ang II was attenuated by PDE5 inhibition. The decline of cell mobility was caused by phosphorylation of TRPC6 at T69 and S321 (T70 and S322 in humans) by PKG. The finding suggested a potential therapeutic method for the treatment of glomerular disease caused by hyperactive TRPC6 [108]. 

### 6.5. Phosphorylation-Induced Trafficking of TRPC6

The relationship between phosphorylation and trafficking of TRPC6 was firstly outlined by Kanda et al. The study showed that the Src family kinase catalyzed the phosphorylation of TRPC6 at Y284, facilitating physical interaction between TRPC6 and phospholipase C (PLC)-γ1. Association between TRPC6 and PLC was a prerequisite for TRPC6 plasma membrane trafficking. Nephrin competitively bound to TRPC6, which decreased its basal activity as a result. The FSGS-causing TRPC6 mutant was found to be insensitive to the suppression of nephrin [109]; therefore, there was excessive Ca^2+^ influx in this mutant. An unbiased phosphoproteomic screen of human TRPC6 was conducted by Hagmann et al. in 2018, where several novel phosphorylation sites were identified, including S4, S13, S14, and S814. Among these sites, S14 was proven to be the target of MAPKs and proline-directed kinases such as cyclin-dependent kinase 5 (Cdk5). Phosphorylation at this amino acid increased surface expression and constitutive activity of TRPC6 [110]. In fact, S814 of TRPC6 in mouse spleen and lung had also been reported by Huttlin et al. in 2010 in attempt to setup a tissue-specific map of phosphoproteome in mouse [111]. Later in 2011, Bousquet et al. also reported this PTM in HEK-293 under unstimulated conditions, and found that this PTM exerts no effects on TRPC6’s activity [112].

### 6.6. Phosphorylation-Mediated Protein-Protein Interaction of TRPC6

In neuronal PC12D cells, M1 muscarinic acetylcholine receptor (mAChR), PKC and TRPC6 were found to form a transient ternary complex after stimulation of carbachol, where PKC phosphorylated the S768 of TRPC6. This phosphorylated TRPC6 was required for the recruitment of immunophilin FKBP12, calcineurin and calmodulin. The phosphatase calcineurin subsequently dephosphorylated TRPC6 and disrupted its interaction with M1-mAChR. Elaboration on these molecular events provided an insight into the complex regulation of channel activation and inhibition [113]. 

### 6.7. PTM of TRPC6 Discovered by High-Throughput Experiments

**Figure 5:**
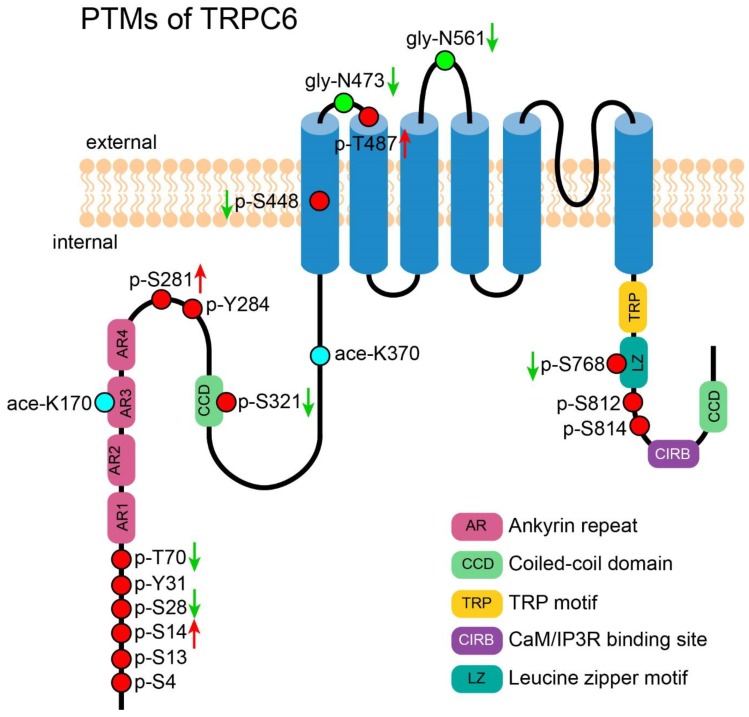
PTMs of TRPC6. A cartoon depicting TRPC6 with its important motifs and PTMs (those exact positions had been reported). PTMs are presented in circles. Red circles represent the reported phosphorylation sites of TRPC6, which are labeled in the format of a “p-amino acid-position”. Green circles represent the reported N-glycosylation sites, which are labeled in the format of a “gly-amino acid-position”. Cyan circles represent the reported acetylation sites, which are labeled in the format of a “ace-amino acid-position”. The green arrows near the labels denote the PTMs which decrease the channel’s activity. The red arrows near the labels denote the PTMs which increase the channel’s activity.

High-throughput studies from mass spectrometry identified a multitude of TRPC6 PTM sites. Although most sites overlapped with those reported by other studies as reported in previous sections, several unidentified sites were also revealed. Y31 was found in untreated non-small-cell lung cancer cell lines in a large-scale search for substrates of oncogenic receptor tyrosine kinases (RTKs) [114]. In a comprehensive phosphoproteomics survey on many different organs of mice, S812 was identified in the spleen [111]. Further studies are needed to examine the role of those PTMs in TRPC6. Figure 5 shows an overview of all PTMs of TRPC6.

## 7. PTM of TRPC7

### 7.1. Phosphorylation of TRPC7

TRPC7 is also the least studied channel among all TRPC members with regard to PTMs. In HEK-293 cells, T15 of TRPC7 was reported to be phosphorylated by cGK-Iα and reduced carbachol-induced Ca^2+^ influx. Both cGK-Iα and cGK-Iβ were found to physically bind to N-terminal ankyrin repeat region of TRPC7 and execute the phosphorylation. Subsequent decline of Ca^2+^ permeation through phosphorylated TRPC7 led to a decrease in the phosphorylation of cAMP response element-binding protein (CREB) and reduced the activity of CREB [115]. S714 of TRPC7 was reported to be phosphorylated by PKC and this modification inhibited the channel’s activity in fibroblast cells. Cell surface proteoglycan syndecan-4 was the upstream molecule coupling extracellular protein ligands of cell surface polysaccharides (such as heparan sulfate) to the activation of PKC. PKC subsequently phosphorylates TRPC7 and negatively regulates its activity. By this way, the intracellular Ca^2+^ level could be well-controlled to ensure the organization of cytoskeleton and junctions required for normal fibroblastic phenotype [116]. 

### 7.2. PTM of TRPC7 Discovered by High-Throughput Experiments

**Figure 6:**
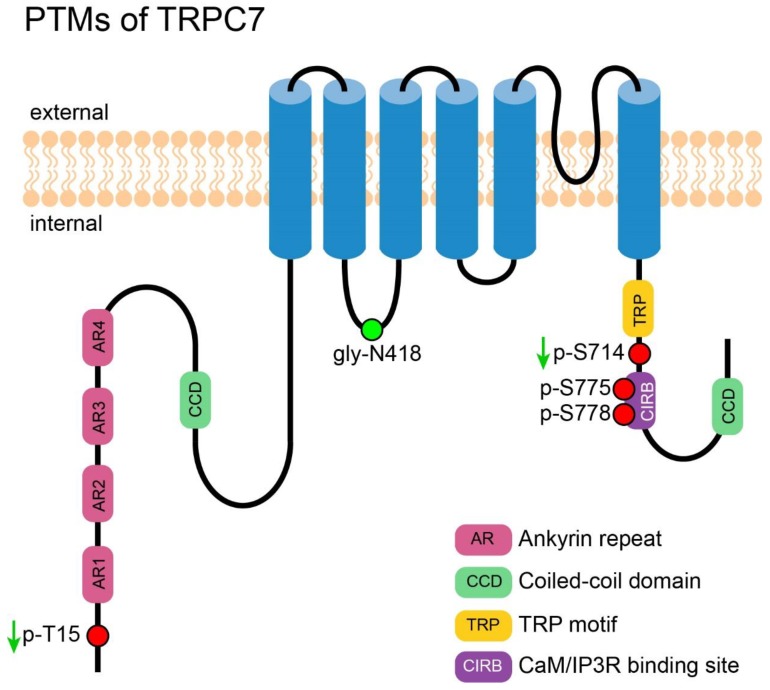
PTMs of TRPC7. A cartoon depicting TRPC7 with its important motifs and PTMs (those exact positions had been reported). PTMs are presented in circles. Red circles represent the reported phosphorylation sites of TRPC7, which are labeled in the format of a “p-amino acid-position”. The green circle represents the reported N-glycosylation site, which is labeled in the format of a “gly-amino acid-position”. The green arrows near the labels denote the PTMs decrease the channel’s activity.

Three novel PTM sites of TRPC7 were identified from high-throughput experiments, including phosphorylation at S775, T778 [117], and N-glycosylation at N418 [118]. It is worthy of note that N418 is predicted to be located at the 2nd cytosolic segment of TRPC7; however, it is rather unusual for a glycosylation to be detected intracellularly, since glycosylation is expected to occur in extracellular segments of a membrane protein. Further validation is needed to confirm the modification on this site. Figure 6 shows an overview of all PTMs of TRPC7.

Table 1 and Table 2 document all reported PTMs in traditional experiments and in high throughput studies, respectively.

## 8. TRPC6 Mutation and Focal Segmental Glomerulosclerosis (FSGS)

### 8.1. Introduction of FSGS

FSGS is a podocyte disease with clinical features of edema, proteinuria, hypoalbuminemia, and hyperlipidemia. It accounts for 20% of nephrotic syndrome and 75% of steroid-resistant nephrotic syndrome (SRNS) in children [123]; it is also the leading glomerular cause of end-stage renal disease (ESRD) in the United States [124]. FSGS is a progressive glomerular disease. The lesion starts from podocyte foot process effacement, is followed by podocyte detachment and loss, and subsequently by glomerular capillaries sclerosis. In the early stage, only a minority of glomeruli undergoes glomerulosclerosis, and the sclerosis shows a segmental pattern. With progression, more widespread and global glomeruli are involved in the pathological change, ending in ESRD [125]. 

FSGS can be classified into five forms, including primary (idiopathic) and secondary (inherited, virus associated, drug induced, and adaptive) FSGS. Several genes have been found to underlie the inherit form of FSGS; the spectrum of these genes is wide, ranging from those responsible for slit diaphragm architecture to those responsible for cytoskeleton, including NPHS1, NPHS2, NPHS3, CD2AP, Myo1E, ACTN4, INF2, and TRPC6 [126]. Since there is widespread expression of TRPC6 in different types of tissue, but most of its mutants are just found to be associated with FSGS, it is reasonable to underscore its unique function in the kidney.

### 8.2. Overview of TRPC6 Mutations and SNP

**Figure 7:**
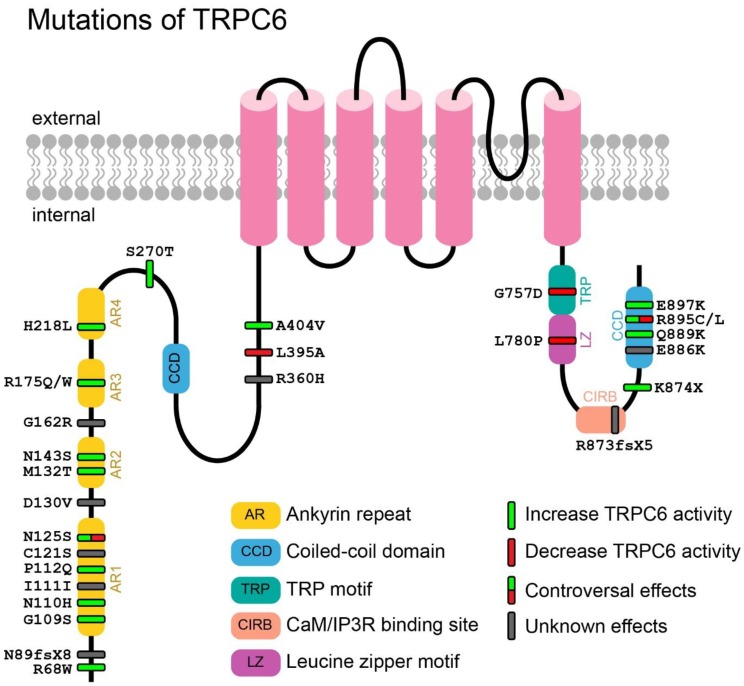
Mutations that cause changes in TRPC6 proteins. A cartoon depicting TRPC6 with its important motifs and mutations (missense substitution, frame shift and deletion). Mutations are presented in bars. Green bars denote GOF mutations, while red bars denote LOF mutations. Gray bars denote mutations with unknown effects (function of which has not been studied), while bars with half green and half red colors denote mutations with controversial effects (controversial reports are found).

Mutations of TRPC6 had been found to cause an inherited form of FSGS since 2005 [127,128]. From that on, novel mutations of TRPC6 relevant to FSGS was increasingly reported, and there are so far 26 mutation sites being reported to date. Figure 7 shows an overview of all mutations that cause change(s) in TRPC6 protein. 

Mutations of TRPC6 can be classified into three categories, including synonymous (one site), missense (22 sites, the most case) and nonsense (three sites) mutations. At the mRNA level, these mutations are mainly located in exon 2 (15 sites) and 13 (seven sites). At the protein level, half of them are located in or near the ankyrin repeat region (13 sites) on the N-terminus, five sites in the coiled-coil domain on the C-terminus, two sites in or near the CIRB domain, one site in the TRP motif, and one site in the leucine zipper motif. Both the ankyrin repeat region and the coiled-coil domain are reported to be important for TRPC6 multimerization and interaction with other proteins [129,130,131], while the CIRB domain is critical for channel activity regulation involving CaM/IP_3_R [132,133,134]. The leucine zipper motif is also reported to be important for the regulation of TRPC6 [135]. In addition, the TRP motif, which is a highly conserved region among all TRP channels, is essential for TRP channel activation [135,136]. Sitting in these important domains, the reported mutations do change the properties of TRPC6 in terms of activity, kinetics, and subcellular location. G109S, N110H, Q889K, R895C, and E897K were reported to increase the basal activity of TRPC6 [137]; N143S, H218L and A404V augmented the maximum influx of Ca^2+^ [137]; M132T, S270T and K874X caused delayed inactivation of TRPC6 [128,138]; R68W, P112Q enhanced the cell surface expression of the channel [127,139]. A recent study of TRPC6 protein structure from cryo-EM has provided a more direct and detailed mechanism of alterations caused by these mutations [140]. G109S, P112Q, N143S, R895C, and E897K, which are located at buried interface between the ankyrin repeats and the vertical helix, are not accessible from the outside. They were therefore speculated to influence the internal movement of the ankyrin repeats and the coiled-coil helix instead of influencing the interaction of TRPC6 with other proteins. M132T, located at the joint interface of two adjacent subunits, was speculated to affect the intersubunit interaction [140].

Although most (17 out of 22) of the missense mutants are gain-of-function (GOF) mutants, five mutants with loss-of-function (LOF) phenotype were also found; they are N125S, L395A, G757D, L780P, and R895C [137]. Riehle et al. proposed that these LOF mutants may cause early onset of FSGS, while GOF mutants may lead to late onset of FSGS. Notably, in contrast to Riehle et al.’s report, N125S and R895C were reported to increase the TRPC6 activity by Gigante et al. and Reiser et al., respectively [128,141]. The discrepancy may due to different testing methods. Riehle et al. measured the activity of TRPC6 mutant by Fura-2 based calcium imaging, while Reiser et al. examined the channel current by whole cell patch clamp; in the latter method, cations other than Ca^2+^ may pass through the TRPC6 mutants and contribute to a larger whole cell current than that recorded in WT TRPC6. 

Besides all these mutations, there was a report of SNP (rs3824934) in the promoter region of TRPC6. This SNP increases TRPC6 expression and leads to steroid-resistant nephrotic syndrome (SRNS) in Chinese children [142]. 

### 8.3. Mechanism of TRPC6-Mutant Induced FSGS

Although TRPC6 is not the only gene whose mutations are attributed to the FSGS, podocyte-specific overexpression of WT or two GOF mutants, P111Q and E896K (corresponding to P112Q and E897K of human TRPC6), was sufficient to cause FSGS in mice, which means TRPC6 is essential for FSGS [143]. Because most TRPC6 mutants possess GOF phenotypes, the rise of TRPC6 activity will elevate cytosolic [Ca^2+^]_i_ and subsequently trigger a wide array of signaling pathways.

Activation of calcineurin was found in TRPC6 mutants P112Q, R895C and E897K. Activated calcineurin may lead to degradation of synaptopodin which regulates the cytoskeleton of podocytes; activated calcineurin would also potentiate basal and GPCR-activated NFAT-mediated transcription, which lead to the dysfunction of podocytes [144]. Chiluiza et al. reported that R895C mutation activated ERK1/2 via two different pathways: a cell-autonomous, EGF receptor-independent pathway and a non-cell-autonomous, EGF receptor-dependent pathway [145]. Activation of ERK may promote nuclear translocation of NF-κB and induce apoptosis of podocytes [146]. TRPC6 was shown to be expressed in both podocyte cell body and foot progress and interact with podocin and nephrin [128]. Nephrin was found to competitively bind to phosphorylated-TRPC6-PLC complex and inhibit the trafficking of TRPC6 to the plasma membrane. But TRPC6 mutants (P112Q, N143S, S270T, R895C, and E897K) showed a weaker interaction with nephrin therefore escaped the inhibition by nephrin, resulting in a rise of cell surface expression and an increased Ca^2+^ influx [109]. According to Verheijden et al.’s report, GOF mutants together with upregulated expression of WT TRPC6 led to an enhancement of calpain and calcineurin activity in podocytes and a decrease of talin-1 expression. As talin-1 is an important bridge between actin cytoskeleton and integrins, the decline of talin-1 hampered the stability of cytoskeleton and led to podocyte injury [147]. Farmer et al. highlighted the association between calpain and TRPC6 in podocytes independent of its ion channel function. TRPC6 was found to act as a scaffold protein that binds to calpain thus affecting the mobility and detachment of podocytes [148]. Mutations located in the coiled-coil domain (K874X, Q889K, R895C/L, and E897K) were found to impair the CaM-mediated Ca^2+^-dependent inactivation of TRPC6, and lead to a sustained Ca^2+^ elevation and disorganization of cytoskeleton [149]. In summary, GOF TRPC6 mutants permeate excessive Ca^2+^, elevate the cytosolic [Ca^2+^], and therefore induce cell death or cytoskeleton disorganization of podocytes, which ultimately leads to FSGS.

## 9. TRPC6 Mutation/SNP and Other Diseases

### 9.1. TRPC6 and Infantile Hypertrophic Pyloric Stenosis (IHPS)

Two SNPs, one in the promoter region of TRPC6 (rs3922961), the other in intron (rs7118839) together with a missense mutation in exon 4 (A404V) were found to underlie infantile hypertrophic pyloric stenosis (IHPS) [150]. IHPS is the most common inherited cause of astrointestinal obstruction in newborn babies with a higher prevalence in males. Hypertrophic smooth muscle of pylorus causes the gastric outlet obstruction and leads to vomiting, weight loss and dehydration in IHPS patients. Although functional characterization of these mutations was absent from the original report, the mutant A404V was later demonstrated to have an increased Ca^2+^ conductance [137]. The SNP in the promoter may affect TRPC6 expression level and the one in intron may affect the splicing efficiency of TRPC6. Although detailed effects of these SNPs need further validation, this discovery provided a new clue for the inherited cause of IHPS.

### 9.2. TRPC6 and Idiopathic Pulmonary Arterial Hypertension (IPAH)

Another SNP in the TRPC6 promoter region (rs3824934) was revealed to cause idiopathic pulmonary arterial hypertension (IPAH) via facilitating the binding of NF-κB to the promoter and enhancing the expression of TRPC6. Increased TRPC6 expression augmented the cytosolic Ca^2+^ in pulmonary arterial smooth muscle cells (PASMCs), which in turn caused contraction and proliferation of PASMCs, leading to the hypertension [151].

### 9.3. TRPC6 and Neuropsychiatric Manifestations (NPSLE)

Patients with systemic lupus erythematosus (SLE) carrying a SNP in TRPC6 intron (rs7925662) was found to be prone to develop neuropsychiatric manifestations (NPSLE). Increased TRPC6 activity was found in peripheral blood mononuclear cells (PBMCs) in SLE patients, which rendered PBMCs less resistant to apoptosis and secreted less interleukin-4 [152].

## 10. TRPC6 and Chronic Fatigue Syndrome (CFS)

A SNP found in TRPC6 intron (rs11224816) together with SNPs in other TRP channels and acetylcholine receptor were suggested to be associated with Myalgic Encephalomyelitis (ME)/Chronic Fatigue Syndrome (CFS) [153]. CFS/ME is a severe systemic illness characterized by impairment of physical activity and debilitating fatigue. It is relevant to the disorder of endocrine, cardiovascular, gastrointestinal, neuro, and immune systems [154]. To date, an efficient and defined diagnostic method for this illness is still absent, the finding of related SNPs may provide a potential screening method for the disease.

## 11. Mutation/SNP of Other TRPC Channels and Corresponding Diseases

### 11.1. TRPC3 and Inherited Cerebellar Ataxia

Inherited cerebellar ataxias are a class of neurodegenerative disorders featured with impaired balance and coordination. Becker et al. discovered a missense mutation of TRPC3 (T635A) which resulted in an alteration of TRPC3 gating in an ataxia animal model, the moonwalker (Mwk) mouse [155]. T635 was a putative phosphorylation site of PKC which negatively regulated the TRPC3 activity. Mutation of this residue into alanine abolished PKC-mediated phosphorylation, leading to an elevation of TRPC3 activity. Loss of control in TRPC3’s activity hampered the growth and differentiation of Purkinje cells and finally led to ataxia.

### 11.2. TRPC4 and Myocardial Infarction (MI)

Interestingly, a missense SNP of TRPC4 (I957V) was found to reduce the risk of myocardial infarction (MI) in diabetic patients [156]. Substitution of isoleucine with less bulky valine allowed tyrosine kinase to approach T959 easier and formed a firmer interaction, as a result, promoted the phosphorylation of mutant TRPC4 and facilitated plasma membrane insertion of the channel. The TRPC4 mutant, with higher constitutive activity, permeated more Ca^2+^ in endothelium to generate more NO. The resulting vasorelaxation induced by NO will thereby help to attenuate the effects of MI.

### 11.3. TRPC4/7 and Lung Cancer

Two intronic SNPs of TRPC4 (rs9547991 and rs978156) and one intronic SNP of TRPC7 (rs11748198) were consistently found to be associated with lung cancer in Chinese patients [157]. Although a detailed mechanism for this remains unconfirmed, Zhang et al. suggested that combined variations were more detrimental than any individual variant and underscored the significance of taking multiple SNPs into consideration for a disease assessment.

Table 3 documents all the reported mutations and SNP sites in TRPCs.

## 12. Concluding Remarks

**Figure 8:**
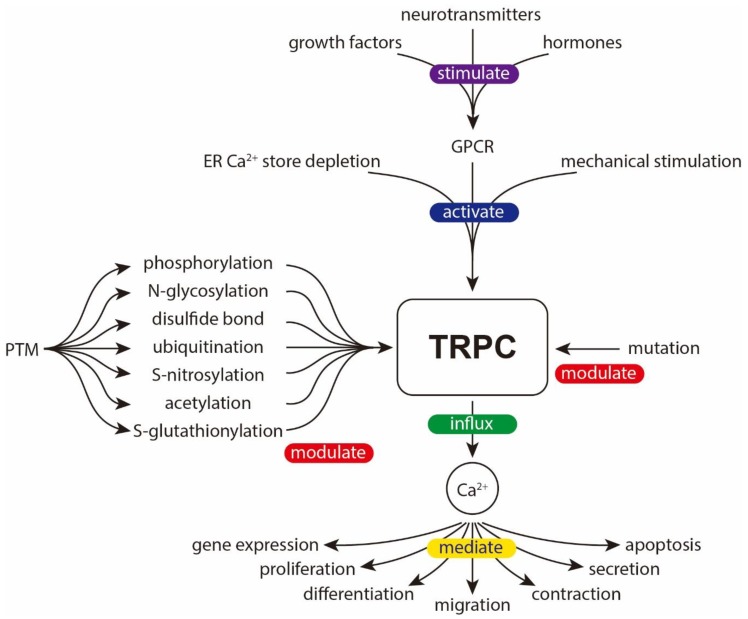
Overview of the signal transduction through TRPC channels. TRPC can be activated by endoplasmic reticulum (ER) Ca^2+^ store depletion, GPCRs (sensing extracellular stimuli such as growth factors, neurotransmitters, hormones etc.), and mechanical stimulation. The activity of TRPC can be modulated by various PTMs and mutations. The downstream effect of TRPC activation is mainly mediated by Ca^2+^ influx. As an important second messenger, Ca^2+^ may trigger a wide array of signaling pathways and lead to various biological processes including change in gene expression, proliferation, differentiation, migration, contraction, secretion, and apoptosis.

TRPCs are versatile channels widely expressed in different types of cells and tissues; they sense various kinds of extracellular stimuli and relay them into different output responses. PTM is an important strategy used to modulate the channel’s properties, therefore allowing the cells to better meet different physiological requirements. An equally important aspect is the natural mutations found in TRPCs. These mutations may generate GOF/LOF mutants and cause maladaptive responses and/or inherited diseases. Figure 8 shows an overview of the signal transduction through TRPC channels. 

TRPCs can be covalently modified by several types of PTM. In general, phosphorylation is the most common one, it may enhance or decrease the channel’s activity, affect the trafficking of the channel or the interaction of TRPCs with other proteins. According to the few reports that documented N-glycosylation of TRPCs, N-glycosylation tends to reduce the channel activity. A disulfide bond may help to stabilize the pore architecture and also to confer the sensitivity of the TRPCs to reducing reagents. TRPC5 can be subjected to S-nitrosylation or S-glutathionylation; this sensitivity to oxidation stress allows a brand-new regulation pathway for this channel. Ubiquitination causes internalization but not degradation of TRPC4. Acetylation is found in TRPC6, but its function is still unknown. 

For natural mutations reported in TRPC channels, TRPC6 mutants and the related disease FSGS are the best studied. Most TRPC6 mutants are characterized to have GOF phenotype; they elevate the cytosolic Ca^2+^, and subsequently cause cell death and/or cytoskeleton disorganization of podocytes, resulting in FSGS. Another well-studied mutation is the TRPC3 mutant in moonwalker mice. The mutation also results in a GOF phenotype, impairs the growth and differentiation of Purkinje cells and leads to ataxia. SNPs of TRPC6 are found to be associated with diseases such as IHPS, IPAH, NPSLE and CFS. While most mutations or SNPs are linked to cellular dysfunction, it is intriguing that a missense SNP in TRPC4 has been found to decrease risk of MI in diabetic patients.

The traditional strategy to study PTM of proteins begins with prediction of modification site, followed by site-directed mutagenesis, and then expression and functional characterization. By this method, most PTMs of TRPCs have been identified and studied. Although this method provides the best functional annotation of each PTM, the drawback is that only a certain type of modification in a particular channel can be studied each time. On the other hand, by using the high-throughput, mass spectrometry-based method, a global and unbiased search of multiple types of PTM in a large population of proteins can be conducted. It has been proven that when using the high-throughput method, several novel PTM sites in TRPCs could be identified. The biggest drawback of high-throughput method is the lack of functional annotation of the identified PTM. High throughput methods and traditional methods are complementary to each other and can be used in conjunction to investigate PTMs of TRPCs comprehensively. In the future, it is ideal to screen for changes of PTMs under different physiological or pathological conditions by using the high-throughput method followed by a confirmation and characterization using traditional methods to better understand their influence on the TRPC channel proteins and on the cells.

Recently, the protein structure of TRPC3/4/5/6 have been resolved by cryo-EM with an atomic resolution [81,140,169,170,171]. The detailed 3D structure offers a new way to evaluate potential residues that are critical for the gating and/or permeation of TRPCs. It would also be interesting and fruitful to revisit those missense or nonsense mutations or SNPs from public database, and to speculate and/or to investigate the possible effects of these mutations/SNPs on the TRPC proteins and on the cells.

## Figures and Tables

**Table 1:** PTMs of different members of TRPC as reported by traditional experimental studies.

Channel	Modification	Species and Accession Number	Site	Sequence	Enzyme	Cell	Effect	Reference
TRPC1	Phosphorylation	Human n.a.	S/T/Y	n.a.	PKCα	HUVEC and HMEC	Increased TRPC1 activity, enhanced permeability of endothelial monolayers	[47]
TRPC1	Phosphorylation	Rabbit n.a.	S/T	n.a.	PKC	Rabbit portal vein myocytes	Increased TRPC1 activity, promoted PIP_2_-mediated SOCE	[51]
TRPC1	Phosphorylation	Human P48995.2	S172 T313	SAKNKKDSLRHSRFR SGYRRKPTCKKIMTV	PKG	Porcine coronary arterial smooth muscle cell	Decreased TRPC1 activity, inhibited NO-, PKG-induced smooth muscle hyperpolarization and relaxation	[55]
TRPC1	Phosphorylation	Human	S/T/Y	n.a.	PKCα	HUVEC	Increased TRPC1 activity, promoted monocyte binding to endothelial cells and increased endothelial hyperpermeability	[48]
TRPC1	Phosphorylation	Rabbit n.a.	S/T	n.a.	PKC	Rabbit vascular smooth muscle cell	Increased TRPC1 activity	[52]
TRPC1	Phosphorylation	Human P48995.2	S172 T313	SAKNKKDSLRHSRFR SGYRRKPTCKKIMTV	PKG	Primary mesenteric artery endothelial cells	Decreased TRPC1 activity	[56]
TRPC1	Phosphorylation	Rat	S/T	n.a.	PKCα	INS-1E	Increased TRPC1 activity, promoted insulin secretion	[54]
TRPC3	N-glycosylation	Human Q13507.3	N416 (N418 *)	EGITTLPNITVTDYP	n.a.	COS-M6 and HEK-293	n.a.	[58,59]
TRPC3	Phosphorylation	Human Q13507.3	S712	PPFSLVPSPKSFVYF	PKC	HEK-293	Decreased TRPC3 activity	[63]
TRPC3	Phosphorylation	Human Q13507.3	T11 S263	SPSLRRMTVMREKGR KNDYRKLSMQCKDFV	PKG	HEK-293	Decreased TRPC3 activity, abolished the SOCE mediated by TRPC3	[62,64]
TRPC3	Phosphorylation	Human Q13507.3	Y49 Y148 Y150 Y226	RFLDAAEYGNIPVVR ELQDDDFYAYDEDGT QDDDFYAYDEDGTRF KGLASPAYLSLSSED	*Src*	HEK	Phosphorylation at Y226 but not others increased TRPC3 activity	[61]
TRPC3	Phosphorylation	Human Q13507.3	T573	LQISLGRTVKDIFKF	PKC	HL-1	Coupled TRPC3 signaling to the activation of the NFAT pathway	[65]
TRPC4	Phosphorylation	Human Q9UBN4.1	Y959 Y972	EEDSSIDYDLNLPDT DTVTHEDYVTTRL	*Src*	COS-7	Induced insertion of TRPC4 into plasma membrane and its association with NHERF	[70]
TRPC4	ubiquitination	Mouse Q9QUQ5.2	N terminus	n.a.	AIP4	HEK-293T	Promoted endocytosis of TRPC4 and reduced its basal activity	[75]
TRPC4	Phosphorylation	Mouse Q9QUQ5.2	S688	KMRRKPESFGTIGRR	PKG	HEK-293	Increased TRPC4 activity	[71]
TRPC4	Disulfide bond	Mouse Q9QUQ5.2	C549 C554	TKGLSCKGIRCEKQNN	n.a.	n.a.	Constrained TRPC4 activity	[76]
TRPC5	Phosphorylation	Mouse Q9QX29.1	T972	DGQEEQVTTRL	PKC	HEK-293	Accelerated desensitization of TRPC5, necessary for the association with NHERF	[36,77]
TRPC5	S-nitrosylation	Mouse Q9QX29.1	C553 C558	DEPNNCKGIRCEKQNN	n.a.	HEK, vascular endothelial cells	Increased TRPC5 activity	[82]
TRPC5	Disulfide bond	Human Q9UL62.1	C553 C558	DEPNNCKGIRCEKQNN	n.a.	HEK-293	Constrained TRPC5 activity	[79]
TRPC5	Phosphorylation	Human Q9UL62.1	S794 S796	SGGARAKSKSVSFNL	PKA	HEK-293	Decreased TRPC5 activity	[78]
TRPC5	S-glutathionylation	Human Q9UL62.1	C176 C178	RPHQIRCNCVECVSS	n.a.	HEK-293, Clonal striatal cells	Increased TRPC5 activity, activated the calmodulin- dependent protein kinase and calpain-caspase pathway	[87]
TRPC5	Disulfide bond	Human Q9UL62.1	C553 C558	DEPNNCKGIRCEKQNN	n.a.	HEK-293	Stabilized TRPC5 multimerization, promoted its plasma membrane insertion	[80]
TRPC5	Disulfide bond	Mouse Q9QX29.1	C553 C558	DEPNNCKGIRCEKQNN	n.a.	n.a.	Essential for TRPC5 activation	[81]
TRPC6	N-glycosylation	Mouse Q61143.1	N472 N560	EGTKLLPNETSTDNA AQSIIDANDTLKDLT	n.a.	COS	n.a.	[6]
TRPC6	N-glycosylation	Rat Q99N78.1	N711	YVLYGVYNVTMVIVL	n.a.	COS	n.a.	[88]
TRPC6	N-glycosylation	Human Q9Y210.1	N473 N561	EGTKLLPNETSTDNA AQSIIDANDTLKDLT	n.a.	HEK-293	Decreased TRPC6 constitutive activity	[59]
TRPC6	Phosphorylation	n.a.	Y	n.a.	*src*	COS-7	Increased TRPC6 activity	[96]
TRPC6	Phosphorylation	Rat Q99N78.1	S768	VPFNLVPSPKSLLYL	PKC	PC12D	Decreased TRPC6 activity, required for protein complex formation upon activation of the M1 muscarinic acetylcholine receptor	[113]
TRPC6	Phosphorylation	Mouse Q61143.1	T69	RLTHRRQTILREKGR	PKG	HEK-293 and A7r5	Decreased TRPC6 activity	[86]
TRPC6	Phosphorylation	Mouse Q61143.1	S448	KFVAHAASFTIFLGL	PKC	HEK-293T and A7r5	Decreased TRPC6 activity, reduced vasopressin-induced Ca^2+^ entry	[107]
TRPC6	Phosphorylation	Mouse Q61143.1	T69	RLTHRRQTILREKGR	PKG	NRVM	Decreased TRPC6 activity, suppressed NFAT activation, prevented cardiac hypertrophy	[100,102]
TRPC6	Phosphorylation	Human Q9Y210.1	T70 S322	RLAHRRQTVLREKGR KNDYKKLSMQCKDFV	PKG	NRVM and AMVM	Decrease TRPC6 activity, suppressed NFAT activation, prevented cardiac hypertrophy	[101]
TRPC6	Phosphorylation	Mouse Q61143.1	T69	RLTHRRQTILREKGR	PKA	RAoSMC	Decreased TRPC6 activity, attenuated angiotensin II-induced vasoconstriction	[104]
TRPC6	Phosphorylation	Mouse Q61143.1	S814	KKFGISGSHEDLSKF	n.a., but not casein kinase II	HEK-293	No effect on TRPC6 activity	[112]
TRPC6	Phosphorylation	Mouse Q61143.1	S281	NAYKGLASPAYLSLS	ERK1/2	HEK-293 and Rat Glomerular mesangial cell	Increased TRPC6 activity, necessary for cAMP-induced Ca^2+^ influx	[98]
TRPC6	Phosphorylation	Mouse Q61143.1	Y284	KGLASPAYLSLSSED	Src	HEK-293T, podocyte	Enhanced plasma membrane trafficking of TRPC6	[109]
TRPC6	Phosphorylation	Mouse Q61143.1	S28 T69	AGTRRNESQDYLLMD RLTHRRQTILREKGR	PKA	HEK-293	S28 but not T69 decreased TRPC6 activity	[105]
TRPC6	Phosphorylation	Mouse Q61143.1	T69	RLTHRRQTILREKGR	cGKI	MLEC and HDMEC	Decreased TRPC6 activity, prevented endothelial cell hyperpermeability responses to hyperforin	[106]
TRPC6	Phosphorylation	Mouse Q61143.1	T487	RQLFRMKTSCFSWME	CaMKII	HEK293 and A7r5	Increased TRPC6 activity, enhanced arginine vasopressin-induced cation current in smooth muscle cells	[97]
TRPC6	Phosphorylation	Mouse Q61143.1	T69 S321	RLTHRRQTILREKGR KNDYKKLSMQCKDFV	PKG	Murine podocyte	Decreased TRPC6 activity, reduced podocyte motility	[108]
TRPC6	Phosphorylation	Dog	T	n.a.	n.a.	Canine heart left ventricle	Decreased TRPC6 activity, underpinned the tadalafil-caused delay of dystrophic cardiomyopathy onset	[103]
TRPC6	Phosphorylation	Human Q9Y210.1	S4 S13, S14 S814	MSQSPAFGPRR AFGPRRGSSPRGAAG KKLGILGSHEDLSKL	MAPK Cdk-5	HEK-293T	S14 enhanced TRPC6 plasma membrane trafficking and constitutive activity	[110]
TRPC6	N-glycosylation	Human Q9Y210.1	N473 N561	EGTKLLPNETSTDNA AQSIIDANDTLKDLT	n.a.	TRex293, podocyte	Required for channel activity, ERK activation and cytotoxicity of the GOF TRPC6 mutant (R895C)	[89]
TRPC7	Phosphorylation	Mouse Q9WVC5.1	T15	KNMQRRHTTLREKGR	cGKI	COS-7 and HEK-293T	Decreased TRPC7 activity	[115]
TRPC7	Phosphorylation	Mouse Q9WVC5.1	S714	APFNLVPSPKSFYYL	PKC	MEF	Decreased TRPC7 activity, regulated cytoskeleton organization and myofibroblast phenotype	[116]

* Denotes PTM sites reported in the original papers but they are actually the same sites as indicated in the table. INS-1E: rat insulinoma cells. HUVEC: human umbilical vein endothelial cells. HMEC: human microvessel endothelial cells. COS: African green monkey kidney fibroblast-like cell line. HEK: human embryonic kidney cell line. HL-1: murine atrial myocytes. Hela: human cervix epithelia cell line. PC12D: rat adrenal gland cell line. NRVM: neonatal rat ventricular myocyte. AMVM: adult mouse ventricular myocyte. RAoSMC: rat aortic smooth muscle cell. MLEC: mouse microvascular lung endothelial cell. HDMEC: human dermal microvascular endothelial cell. Cdk-5: cyclin-dependent kinase 5. MEF: murine embryonic fibroblasts. A7r5: vascular smooth muscle cell line.

**Table 2:** PTMs of different members of TRPC as reported by high throughput studies.

Channel	Modification	Species and Accession Number	Site	Sequence	Cell/Tissue	Remark	Reference
TRPC1	Phosphorylation	Human P48995.1	Y368	WPVLSLCYLIAPKS	Hep3B and MHCC97H	Found in nonmetastatic instead of metastatic human hepatocellular carcinoma cells	[57]
TRPC3	N-glycosylation	Mouse Q9QZC1.1	N404	EGITTLPNITVIDYP	Mouse brain	Detected only in brain	[118]
TRPC3	Phosphorylation	Human Q13507.3	S843	LSEKLNPSMLRCE	HeLa	Phosphorylation of TRPC3 decreased after mammalian target of rapamycin (mTOR) inhibition	[69]
TRPC3	Phosphorylation	Mouse B1ATV3.1	S807	LELGMGNSKSRLNLF	Mouse brain	n.a.	[66]
TRPC3	Phosphorylation	Mouse B1ATV3.1	S56	PCPRAPPSPGPDASS	Mouse brain synaptic membrane	n.a.	[67]
TRPC3	Phosphorylation	Rat Q9JMI9.1	S801	EIKQDISSLRYELLE	Rat multiple organs	Detected in intestine	[68]
TRPC4	Phosphorylation	Human Q9UBN4.1	S193	DVDSLRHSRSRLNIY	Hela	n.a.	[73]
TRPC4	Phosphorylation	Mouse Q9QUQ5.2	S688 T691, S875 T879	KMRRKPESFGTIGRR RKPESFGTIGRRAAD SSIDYDLSPTDTAAH YDLSPTDTAAHEDYV	HEK-293	n.a.	[72]
TRPC4	Phosphorylation	Human Q9UBN4.1	S955	KHAKEEDSSIDYDLN	Human liver	n.a.	[74]
TRPC6	Phosphorylation	Human Q9Y210.1	T70	RLAHRRQTVLREKGR	Hela	n.a.	[73]
TRPC6	Phosphorylation	Human Q9Y210.1	S13 S14 Y31 T70	AFGPRRGSSPRGAAG RRNESQDYLLMDSEL RLAHRRQTVLREKGR	NCI-H3255	Phosphorylation changed after Gefitinib treatment	[114]
TRPC6	N-glycosylation	Mouse Q61143.1	N472	EGTKLLPNETSTDNA	Mouse brain	Class I N-glycosylated sites, detected only in the brain	[118]
TRPC6	N-glycosylation	Mouse Q61143.1	N560	AQSIIDANDTLKDLT	Mouse brain, liver, plasma	Class II N-glycosylated sites, detected in multiple organs	[118]
TRPC6	Phosphorylation	Mouse Q61143.1	S812 S814	KKFGISGSHEDLSKF	Mouse multiple organs	S812 was detected in the spleen. S814 was detected in the spleen and lung	[111]
TRPC6	Phosphorylation	Mouse Q61143.1	S814	KKFGISGSHEDLSKF	Mouse spleen	n.a.	[119]
TRPC6	Acetylation	Rat Q99N78.1	K170 K370	ALLLAISKGYVRIVE SRLKLAIKYEVKKFV	Rat multiple organs	K170 was detected in muscles. K370 was detected in the brain, heart, and stomach	[91]
TRPC6	Phosphorylation	Rat Q99N78.1	S4 S814	MSQSPGFVTRR KKFGILGSHEDLSKF	Rat multiple organs	S4 was detected in the lung and spleen. S814 was detected in the spleen, thymus, lung, and blood	[68]
TRPC6	Phosphorylation	Human Q9Y210.1	S815	KKLGILGSHEDLSKL	Colorectal cancer samples	n.a.	[120]
TRPC6	Phosphorylation	Human Q9Y210.1	S815	KKLGILGSHEDLSKL	Non-small cell lung cancer tumors	n.a.	[121]
TRPC6	Phosphorylation	Mouse Q61143.1	S814	KKFGISGSHEDLSKF	Min6	n.a.	[122]
TRPC7	Phosphorylation	Human Q9HCX4.1	S775 T778	LTANNTLSKPTRYQK	HEK	n.a.	[117]
TRPC7	N-glycosylation	Mouse Q9WVC5.1	N418	EGVKTLPNETFTDYP	Mouse brain	Class II N-glycosylated sites, detected only in the brain	[118]

Hep3B: nonmetastatic human hepatocellular carcinoma cell line. MHCC97H: highly metastatic human hepatocellular carcinoma cell line. HEK-293: human embryonic kidney cell line. Hela: human cervix epithelia cell line. NCI-H3255: non-small-cell lung cancer cell lines. Min6: mouse Pancreas Beta Cell line.

**Table 3:** Natural mutations and SNPs of different members of TRPC.

Channel	cDNA and Protein Accession Numbers	Position	Nucleotide Change	Amino Acid Change	Effect	Disease	Reference
TRPC3	NM_019510 NP_062383	Exon	1903A>G	T635A	Increased TRPC3 activity	Hereditary cerebellar ataxias	[155]
TRPC4	NM_016179 NP_057263	Exon	2869A>G (3104A>G *)	I957V	Increased TRPC4 activity	Reduced risk of MI	[156]
TRPC4	NM_016179 NP_057263	Intron	rs9547991: NG_029849.2:g.232148A>G	No change	n.a.	Lung cancer	[157]
TRPC4	NM_016179 NP_057263	Intron	rs978156: NG_029849.2:g.172052G>A	No change	n.a.	Lung cancer	[157]
TRPC6	NM_004621 NP_004612	Exon	202C>T	R68W	Increased plasma membrane trafficking and activity of TRPC6	Proteinuria and FSGS	[139]
TRPC6	NM_004621 NP_004612	Exon	265delA	S89NfsX8	n.a.	FSGS	[158]
TRPC6	NM_004621 NP_004612	Exon	325G>A	G109S	Increased basal and maximum activity of TRPC6	FSGS	[137,159]
TRPC6	NM_004621 NP_004612	Exon	328T>G	N110H	Increased basal and maximum activity of TRPC6	FSGS	[137,160]
TRPC6	NM_004621 NP_004612	Exon	333C>T	I111I	n.a.	Proteinuria and FSGS	[161]
TRPC6	NM_004621 NP_004612	Exon	C335>A	P112Q	Increased plasma membrane trafficking, basal, and maximum activity of TRPC6	FSGS	[127]
TRPC6	NM_004621 NP_004612	Exon	362G>C	C121S	n.a.	Proteinuria and FSGS	[161]
TRPC6	NM_004621 NP_004612	Exon	374A>G	N125S	Increased/decreased TRPC6 activity	FSGS	[137,141,159]
TRPC6	NM_004621 NP_004612	Exon	389A>T	D130V	n.a.	Proteinuria and FSGS	[161]
TRPC6	NM_004621 NP_004612	Exon	395T>C (495T>C *)	M132T	Delayed inactivation, increased basal and maximum activity of TRPC6	FSGS	[137,138,158]
TRPC6	NM_004621 NP_004612	Exon	n.a.	N143S	Increased maximum activity of TRPC6	FSGS	[128,137]
TRPC6	NM_004621 NP_004612	Exon	484G>C	G162R	n.a.	Proteinuria and FSGS	[161]
TRPC6	NM_004621 NP_004612	Exon	524G>A	R175Q	Increased TRPC6 activity	FSGS	[162]
TRPC6	NM_004621 NP_004612	Exon	523C>T	R175W	Increased basal TRPC6 activity	SRNS	[137,163]
TRPC6	NM_004621 NP_004612	Exon	653A>T	H218L	Increased maximum activity and expression of TRPC6	FSGS	[137,141]
TRPC6	NM_004621 NP_004612	Exon	n.a.	S270T	Delayed TRPC6 inactivation	FSGS	[128,164]
TRPC6	NM_004621 NP_004612	Exon	1079G>A	R360H	n.a.	FSGS	[165]
TRPC6	NM_004621 NP_004612	Exon	n.a.	L395A	Decreased TRPC6 activity	SRNS/FSGS	[137,166]
TRPC6	NM_004621 NP_004612	Exon	1211C>T	A404V	Increased maximum activity of TRPC6	IHPS	[137,150]
TRPC6	NM_004621 NP_004612	Exon	2270G>A	G757D	Decreased TRPC6 activity	FSGS	[137,158]
TRPC6	NM_004621 NP_004612	Exon	2339T>C	L780P	Decreased TRPC6 activity	FSGS	[137,159]
TRPC6	NM_004621 NP_004612	Exon	2617_2620 delGATA	D873RfsX5	n.a.	Heterogenous phenotype ranging from asymptomatic minimal change disease to end-stage kidney disease	[25]
TRPC6	NM_004621 NP_004612	Exon	n.a.	K874X	Delayed TRPC6 inactivation	FSGS	[128,138]
TRPC6	NM_004621 NP_004612	Exon	2656G>A	E886K	n.a.	SRNS/FSGS	[167]
TRPC6	NM_004621 NP_004612	Exon	2665C>A	Q889K	Increased basal and maximum activity of TRPC6	FSGS	[137,168]
TRPC6	NM_004621 NP_004612	Exon	n.a.	R895C	Increased TRPC6 expression, increased/decreased TRPC6 activity	FSGS	[128,137]
TRPC6	NM_004621 NP_004612	Exon	2684G>T	R895L	Increased TRPC6 activity	FSGS	[141]
TRPC6	NM_004621 NP_004612	Exon	n.a.	E897K	Increased basal and maximum activity of TRPC6	FSGS	[128,137]
TRPC6	NM_004621 NP_004612	Promoter	rs3922961: NG_011476.2:g.4512A>C	No change	n.a.	IHPS	[150]
TRPC6	NM_004621 NP_004612	Promoter	rs3824934: NG_011476.2:g.5172C>G	No change	Increased TRPC6 expression	IPAH	[151]
TRPC6	NM_004621 NP_004612	Promoter	rs3824934: NG_011476.2:g.5172C>G	No change	Increased TRPC6 expression	SRNS	[142]
TRPC6	NM_004621 NP_004612	Intron	rs7118839: NG_011476.2:g.50182G>A	No change	n.a.	IHPS	[150]
TRPC6	NM_004621 NP_004612	Intron	rs7925662: NG_011476.2:g.51354A>G	No change	n.a.	NPSLE	[152]
TRPC6	NM_004621 NP_004612	Intron	rs11224816: NG_011476.1:g.63374A>G	No change	n.a.	ME/CFS	[153]
TRPC7	NM_020389 NP_065122	Intron	rs11748198: NC_000005.9:g.135575829C>A	No change	n.a.	Lung cancer	[157]

* Denotes the numbering reported in the original paper. (We have standardized the numbering to numbering in coding sequences. Therefore, number ‘1’ represents the nucleotide ‘A’ in the start codon ‘ATG’.) ESKD: end-stage kidney disease. FSGS: focal and segmental glomerulosclerosis. IHPS: infantile hypertrophic pyloric stenosis. IPAH: idiopathic pulmonary arterial hypertension. ME/CFS: myalgic encephalomyelitis/chronic fatigue syndrome. MI: myocardial infarction. NPSLE: neuropsychiatric manifestations. SRNS: steroid resistant nephrotic syndrome.
